# Reduced insulin/insulin-like growth factor signaling decreases translation in *Drosophila* and mice

**DOI:** 10.1038/srep30290

**Published:** 2016-07-25

**Authors:** Paul Essers, Luke S. Tain, Tobias Nespital, Joana Goncalves, Jenny Froehlich, Linda Partridge

**Affiliations:** 1 Max-Planck Institute for Biology of Ageing, Joseph-Stelzmann Str 9b, Cologne D-50931, Germany; 2 Institute of Healthy Ageing, and GEE, UCL, Darwin Building, Gower Street, London WC1E6BT, UK

## Abstract

Down-regulation of insulin/insulin-like growth factor signaling (IIS) can increase lifespan in *C. elegans*, *Drosophila* and mice. In *C. elegans*, reduced IIS results in down-regulation of translation, which itself can extend lifespan. However, the effect of reduced IIS on translation has yet to be determined in other multicellular organisms. Using two long-lived IIS models, namely *Drosophila* lacking three insulin-like peptides (*dilp2-3,5*^*−/−*^) and mice lacking insulin receptor substrate 1 (*Irs1*^*−/−*^), and two independent translation assays, polysome profiling and radiolabeled amino acid incorporation, we show that reduced IIS lowers translation in these organisms. In *Drosophila*, reduced IIS decreased polysome levels in fat body and gut, but reduced the rate of protein synthesis only in the fat body. Reduced IIS in mice decreased protein synthesis rate only in skeletal muscle, without reducing polysomes in any tissue. This lowered translation in muscle was independent of *Irs1* loss in the muscle itself, but a secondary effect of *Irs1* loss in the liver. In conclusion, down-regulation of translation is an evolutionarily conserved response to reduced IIS, but the tissues in which it occurs can vary between organisms. Furthermore, the mechanisms underlying lowered translation may differ in mice, possibly associated with the complexity of the regulatory processes.

Insulin/insulin-like growth factor signaling (IIS) regulates a multitude of processes, including development, growth, metabolism, stress resistance, and reproduction^1^. In addition, mutations that lower IIS can increase lifespan in *C. elegans*, *Drosophila,* mice and, possibly, humans^2^. In *C. elegans,* hypomorphic mutations in the insulin receptor gene *daf-2* result in a robust extension of lifespan^3^. In *Drosophila*, loss of 3 of the 7 insulin-like peptides, (*dilps 2, 3 and 5*) also extends lifespan^4^. Loss of the insulin receptor substrate 1 (*Irs1*) results in lifespan extension in mice and also delays age-related loss of function and pathology^5^. Furthermore, mutations in the human IGF1 receptor that lower IGF1 signaling are enriched in Ashkenazi Jewish centenarians compared to shorter-lived controls^6^, suggesting that the beneficial effect of reduced IIS for longevity may be evolutionarily conserved. However, reduced IIS can also induce several pleiotropic phenotypes, including reduced growth rate, decreased adult body size and reduced fertility. An important challenge is to understand which molecular mechanisms and processes regulate these phenotypes and determine which are relevant specifically to lifespan.

One vital role of IIS is the regulation of translation. Activation of the insulin receptor by binding of insulin/IGF results in recruitment of the insulin receptor substrate (IRS1-4), which activates PI3-kinase and subsequently 3-phosphoinositide-dependent protein kinase 1 (PDK1). PDK1 can regulate translation through two pathways. First, through ribosomal protein S6 kinase (S6K) phosphorylation, PDK1 activates translation initiation factor 4B (eIF4B). Second, PDK1 activates AKT, which in turn activates mechanistic target of rapamycin (mTOR), which then phosphorylates translation initiation factor 4E-binding protein (4E-BP), releasing its inhibition of the translation initiation factor 4E (eIF4E). In addition, mTOR activates S6K, resulting in further activation of eIF4B. Consequently, translation initiation rate and, ultimately, protein synthesis are up-regulated.

Reduced IIS in *C. elegans*, *Drosophila*, and mice results in down-regulation of genes associated with translation^7^ and, accordingly, *C. elegans daf-2* mutants indeed show marked decreases in protein synthesis^8^. Snell dwarf mice, which also show decreased IIS^9^, show a more subtle, but significant, decrease in translation in skeletal muscle and heart^10^. Furthermore, reducing expression of components of the translational machinery can increase lifespan in *C. elegans*^11^,^12^. Tellingly, the lifespan of *daf-2* mutants could not be further extended by depletion of translation-related proteins^11^, suggesting that the role of decreased translation in extension of lifespan was already maximized in the *daf-2* mutant worms. In *Drosophila*, loss of translation initiation factor 5C^13^, as well as inhibition of ribosomal protein S6-kinase and TOR^14^, two regulators of translation, increase lifespan. Mice mutant in ribosomal proteins or translation initiation factors have detrimental phenotypes that would greatly complicate any analysis of their lifespans^15^,^16^. However, loss of ribosomal protein S6K is sufficient to extend lifespan in mice^17^.

How reduced translation increases organismal health during ageing has been discussed extensively elsewhere^11^,^18^,^19^, but the consensus suggests one of two possibilities. First, global reduction in translation may reduce protein folding stress, with the energy so freed redirected towards proteolytic processes, thus preventing the accumulation of misfolded, aggregated or damaged proteins. Second, reducing translation may allow for a switch to translation of specific proteins that are beneficial in the current context^20^,^21^,^22^. A combination of the two mechanisms is also possible.

The need to understand mechanisms of regulation of translation has led to the development of multiple techniques for measuring translation activity and rate. Polysome profiling allows the visualization of mRNA-ribosome complexes through combining density centrifugation and spectrophotometry. The number of ribosomes on mRNAs can be thus quantified, with the association of multiple ribosomes with the mRNA, termed polysomes, potentially indicating a higher level of translation. Conversely, association of an mRNA with a single ribosome, termed monosome, is considered indicative of low translation activity^23^. The overall level of polysomes in a cell can be viewed as a measure of its translational activity. However, the relative levels of monosomes and polysomes reflect a combination of translation initiation, elongation and termination rates. The level of polysomes as a measure of translation takes into account only the number of ribosomes on transcripts, but not the speed with which they move across the transcripts and dissociate from the transcript after their round of translation is complete. A complementary method of measuring translation is to directly assess the rate incorporation of amino acids into polypeptides as they are produced. Typically this is achieved by measuring the incorporation of radioactive amino acids e.g. ^35^S-methionine and ^35^S-cysteine, which over short periods allows a direct assessment of translational rate.

In this study, we used both polysome profiling and ^35^S-incorporation to measure translational activity in multiple tissues from both *Drosophila* and mouse IIS mutants, to determine whether the decrease in protein translation observed upon lowered IIS in *C. elegans* is conserved in *Drosophila* and mice. We used long-lived flies lacking three of the seven *Drosophila insulin-like peptides,* 2, 3 and 5 (hence forth referred to as *dilp2-3,5)*, and long-lived mice which lack the insulin receptor substrate 1 (*Irs1).* In both the *Drosophila dilp2-3,5* mutants and *Irs1*^*−/−*^ mice we found evidence of tissue-specific reductions in translation activity.

## Results

### *Drosophila dilp2-3,5* mutants show tissue-specific reduction of translation

**Figure 1:**
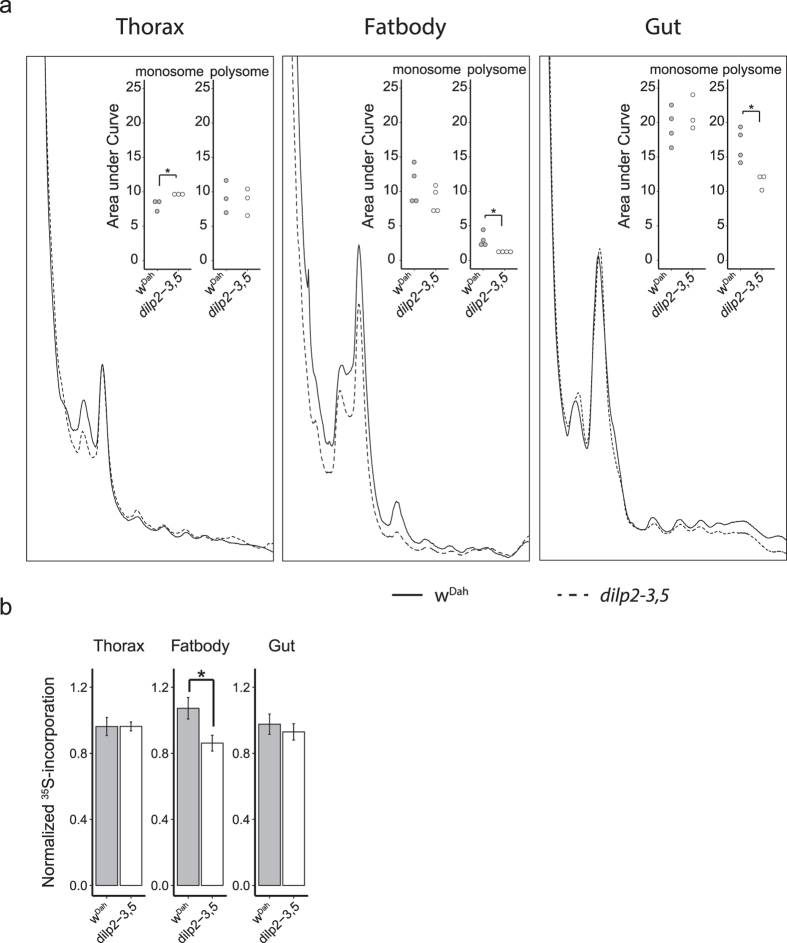
Polysome formation is decreased in the fat body and gut of *dilp2-3,5* mutants. (**a**) Representative polysome profiles of isolated 10 day old *Drosophila* tissues. Insets show the area under the curve measurements for monosomes and combined polysomes. Unpaired student t-test was used to establish significance. *p < 0.05 (n = 4). (**b**) Relative ^35^S counts in dilp2-3,5 mutants for thoraces (n = 17), fat bodies (n = 37), and guts (n = 37). Values were first normalized by protein content, then to batch average. Outliers were removed based on the Grubbs test, unpaired student t-test was used to establish significance. *p < 0.05.

To determine if the translational response to reduced IIS observed in *C. elegans* is evolutionarily conserved, we first assayed translational activity in long-lived *Drosophila dilp2-3,5* mutants. We hypothesized that any IIS-mediated translational response might be tissue-specific, and therefore we examined specific tissues isolated from these flies. We quantified the polysome profiles of the thorax, fat body, gut, and ovary from *dilp2-3,5* and control (*w*^*Dah*^) flies. The fly thorax consists mostly of muscle tissue^24^, while the fat body combines the functions of the mammalian liver and adipose tissue^25^. To quantify differences between profiles we determined the area under the curve (AUC) of both the monosomal peak and the combined polysomal peaks (as depicted in Fig. S1). Individual tissues showed markedly different polysome profiles. Ovaries exhibited more monosomes in comparison to other tissues (Fig. S2), whilst the gut had the highest ratio of polysomes (Fig. 1a). Importantly, quantification revealed that loss of *dilp2-3,5* significantly reduced the ratio of polysomes in the fat body and gut, but not in the thorax or ovaries (Figs 1a and S2). In the fat body the reduced ratio of polysomes was mostly due to reduced numbers of disomes. In contrast, the gut of *dilp2-3,5* mutants showed reduced numbers of higher order polysomes. In addition, we quantified a small, but significant, increase of monosomes in the thorax. Together, these data suggest that the reduced translation in response to reduced IIS is conserved in *Drosophila,* but that the response is highly tissue-specific.

To determine if the reduction in polysome number was correlated with reduced protein synthesis rates, we quantified *de-novo* incorporation of radio-labeled amino acids into proteins in tissues *ex vivo,* focusing on the thorax, fat body, and gut. Isolated tissues were incubated in the presence of radio-labeled methionine and cysteine for 1 hour, to allow sufficient incorporation to occur without saturation (Fig. 1b). In agreement with analysis of the polysome profiles, we detected no differences in ^35^S incorporation levels in the thoraces of *dilp2-3,5* mutants compared to controls. The level of ^35^S incorporation was reduced in fat body of *dilp2-3,5* mutants compared to controls, as suggested by the polysome profile for this tissue. However, contrary to the polysome profile analysis, the gut of *dilp2-3,5* mutants showed no differences in ^35^S incorporation level (Fig. 1b). These data indicate that reduced IIS differentially affects translation initiation and elongation rates in the gut to maintain protein synthesis rate.

### Loss of *Irs1* does not result in reduced polysome formation in mice

**Figure 2:**
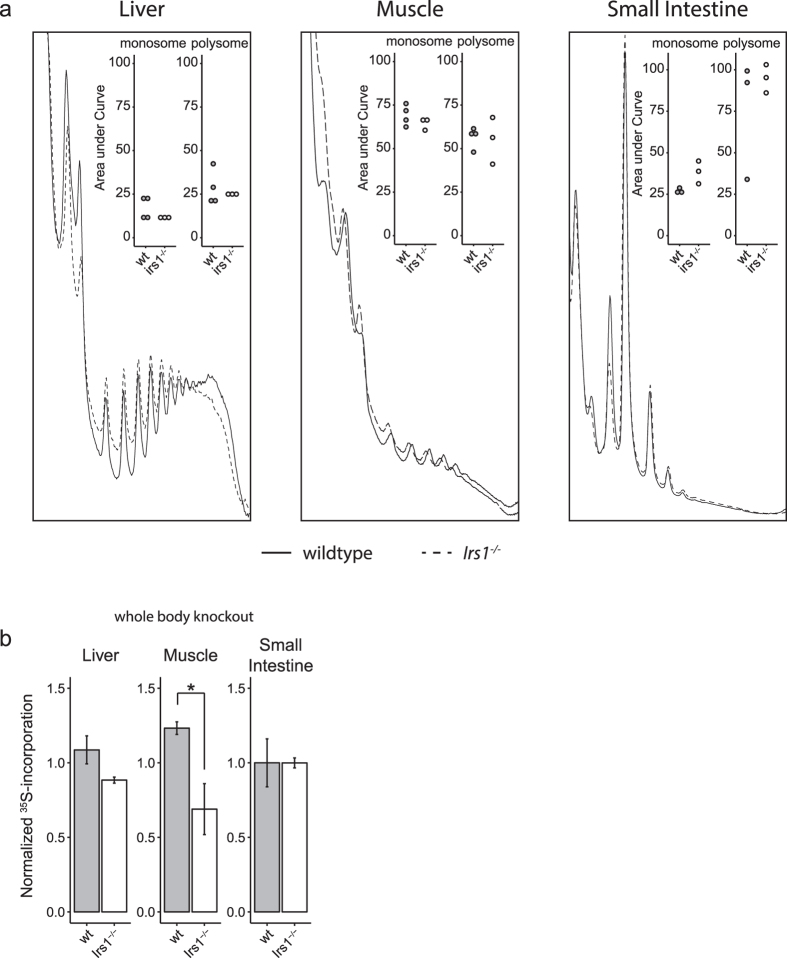
Protein synthesis is reduced in the muscles of *Irs1* mutants, without decreased polysome formation. (**a**) Representative polysome profiles of dissected mouse tissues (74 week old). Insets show area under the curve measurements for monosomes and combined polysomes, wt (n = 4), *Irs1*^*−/−*^ (n = 3). Note that the amount of material loaded varied per tissue, so profiles should not be directly compared between tissues. Unpaired student t-test was used to establish significance. (**b**) Relative ^35^S counts in *Irs1*^*−/−*^ mouse tissues, wt (n = 4), *Irs1*^*−/−*^ (n = 3). For all conditions n = 4 unless stated otherwise. Unpaired student t-test was used to establish significance. *p < 0.05.

In mice, as in *Drosophila,* reduced IIS results in several phenotypes, including growth deficits, altered metabolism and, importantly, increased longevity^5^. To determine if the tissue-specific response of translation to reduced IIS is evolutionarily conserved beyond *Drosophila* and *C. elegans,* we examined polysome profiles in *Irs1* knockout mice. To ensure the most appropriate comparison to our *Drosophila* data, we examined the effect of reduced IIS on translation in skeletal muscle, liver and small intestine. White adipose tissue mass is decreased in *Irs1* knockout mice^5^, greatly complicating any experimental procedures on this tissue, and was therefore omitted from our analyses. As with the *Drosophila* polysome profiles, mouse profiles differed greatly between tissues (Fig. 2a). Loss of *Irs1* did not significantly reduce polysome ratio in any tissue (Fig. 2a). To check the validity of these negative results we examined additional profiles from livers and small intestines (Fig. S3), but failed to detect any significant changes in polysome formation, even after pooling all additional datasets (Fig. S3). In accordance with the polysome profile data, we failed to detect differences in incorporation of ^35^S radio-labeled amino acids into *ex vivo* small intestines (Fig. 2b). However, as in the *Drosophila* gut, polysome formation was not perfectly correlated with the incorporation of ^35^S radio-labeled amino acids into proteins, because we detected a highly significant 44% reduction in incorporation rate in muscle and a 19%, although non-significant, reduction in the liver of *Irs1* knockout mice (Fig. 2b). The sample size may not be large enough to confidently detect a difference of this size in the liver. Conversely, low sample size may also have led to an overestimation of the effect size in the muscle. These findings suggest that, whilst the mechanisms underlying IIS-mediated regulation of translation between *C. elegans, Drosophila*, and mice may differ between translation initiation and elongation, the overall effect on protein synthesis rate may in some tissues be equivalent and lead to a net reduction in translation. The data also highlight the importance of individual tissue-specific responses to reduced IIS, and implicates the muscle, and possibly the liver as important in these responses in mice.

### Loss of *Irs1* reduces translation in muscle non tissue autonomously

We sought to determine if the liver or muscle has a specific role in regulating translation in response to reduced IIS, and if reduced IIS in individual tissues affects the responses of distant tissues non-autonomously. To this end we crossed *Irs1*^*loxP/loxP*^ mice with mice expressing Cre recombinase under the control of the muscle creatine kinase promoter (*Ckmm-Cre*) to generate the *Irs1* skeletal muscle-specific deletion (*Ckmm-Cre::Irs1*^*fl/fl*^). To generate an *Irs1* liver-specific deletion mouse (*Alfp-Cre::Irs1*^*fl/fl*^), we crossed Irs1^loxP/loxP^ mice with mice expressing Cre recombinase under the control of both mouse albumin regulatory elements and α-fetoprotein enhancers (*Alfp-Cre*) (Fig. S4). The polysome profiles of both Irs1^Δskm^ and Irs1^Δliver^ were quantified. Prior to assessing translational status, we confirmed the tissue-specific knockout status of the individual models by qRT-PCR, showing that *Irs1* expression was abolished only in the relevant tissues (Fig. S5a).

**Figure 3:**
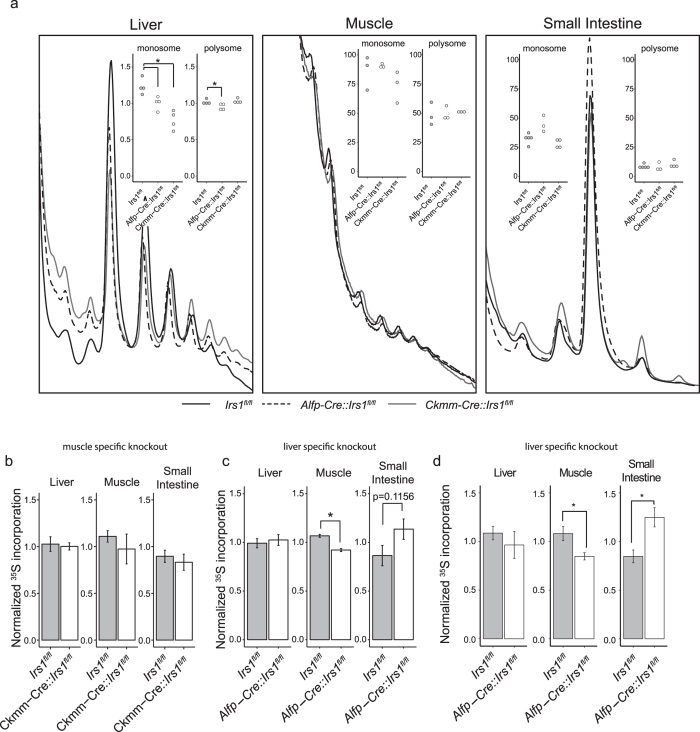
Translation is non-tissue autonomously reduced upon loss of *Irs1*. (**a**) Representative polysome profiles of dissected mouse tissues from muscle specific (*Ckmm-Cre::Irs1*^*fl/fl*^) and liver specific (*Alfp-Cre::Irs1*^*fl/fl*^) *Irs1* knockouts compared to controls (*Irs1*^*fl/fl*^) (n ≥ 3). Insets show area under the curve measurements for monosomes and combined polysomes. Unpaired student t-test was used to establish significance. (**b**) ^35^S counts for muscle specific (*Ckmm-Cre::Irs1*^*fl/fl*^) *Irs1* knockouts and controls (*Irs1*^*fl/fl*^). (**c**) ^35^S counts for liver specific (*Alfp-Cre::Irs1*^*fl/fl*^) *Irs1* knockouts and controls (*Irs1*^*fl/fl*^). Values were first normalized to protein content, then to batch average. (**d**) ^35^S counts in an independent set of Alfp-Cre::Irs1fl/fl mice to verify the findings of Fig. 3C. For all conditions n = 4 unless stated otherwise. Unpaired student t-test was used to establish significance. *p < 0.05, **p < 0.01, ***p < 0.001.

Liver-specific loss of *Irs1* significantly reduced polysome formation in the liver, both at the level of the monosome and the polysome, suggesting a global reduction in translation (Fig. 3a). This tissue-specific loss, however, did not reduce polysome formation in the associated muscle or small intestine of these mice (Fig. 3a). Upon muscle-specific loss of *Irs1* we observed no appreciable effect on polysome formation of either muscle or small intestine but, unexpectedly, we detected a significant reduction in the monosomes in the liver, although the biological relevance of this finding is unclear (Fig. 3a).

In contrast to the reduced translation rate in the muscle upon whole body loss of *Irs1*, loss of *Irs1* specifically in the muscle did not affect translation in any tissue, including the muscle itself (Fig. 3b). Interestingly, liver-specific loss of *Irs1* did not reduce translation rate in the liver, yet led to a significant 22% reduction in translation rate in the muscle and an increase within the small intestine (Fig. 3c), an effect that was reproducible in independent sample sets (Fig. 3d). These findings suggest that liver-specific loss of *Irs1* influences translation in distant tissues as a secondary effect through altered metabolism in the liver, or via an as yet unknown signal. Together, the data indicate that the reduced translational rate observed in the muscle upon whole body loss of *Irs1* is not autonomously mediated by loss of *Irs1* in this tissue. Rather, translation rates can be influenced by distant tissues, with liver-specific loss of *Irs1* regulating translation in the muscle.

## Discussion

The evolutionarily conserved association of reduced IIS and longevity is well established. However, the mechanisms by which reduced IIS extends lifespan remain elusive. Focusing on conserved mechanisms regulated by IIS, and recent studies showing that translation is decreased in *C. elegans* IIS mutants^7^,^8^, we have quantified tissue-specific translation in *Drosophila* and mice, two widely used model organisms in ageing research. We observed highly tissue-specific polysome profiles both in *Drosophila* and mice, as well as highly tissue-specific responses to reduced IIS, emphasizing the need for tissue-specific quantification in such analyses. Lowered IIS significantly reduced the number of polysomes in the gut and fat body of *Drosophila*, but not thorax or ovary. Concordantly, we observed a reduction in translational rate in the fat body, but not in the gut, suggesting that reduced IIS decreases global translation in the fat body whilst changing translation dynamics in the gut to maintain protein output.

In *C. elegans*, the somatic gonad and the intestine make up the majority of the volume of somatic tissue, and thus constitute the major source of protein synthesis. The reduced protein synthesis observed in the *daf-2* mutant is independent of the gonad^8^, suggesting that the IIS-mediated reduction in translation in *C. elegans* occurs mainly in the gut. In line with this, *daf-2* lifespan can be further extended by gonad ablation, suggesting that reduced reproduction is not the downstream mechanism of lifespan extension^26^. However, as both *Drosophila* and *C. elegans* have high fecundity, yolk protein production may constitute a high proportion of protein synthesis activity, and the observed down-regulation of translation may reflect the reduced fecundity observed in *daf-2* worms and *dilp2-3,5* flies^4^,^27^. Furthermore, the gut and fat body are major sites for yolk protein production in *C. elegans* and *Drosophila* respectively^28^,^29^. Nevertheless, the gut and fat body have been implicated as key tissues in the regulation of ageing in *Drosophila*, as a number of manipulations, including reducing IIS, specifically in these tissues is sufficient to increase lifespan^30^,^31^. It is unclear whether this is because gut health is a major determinant of lifespan or due to signaling from the gut and adipose tissue to other tissues, but is likely to involve both^31^. Slowed deterioration of gut health with ageing has been observed in dietary restricted flies^32^, a condition associated with reduced translation^20^, possibly due to a reduction of proteotoxic stress or upregulation of genes involved in defense and maintenance. To our knowledge, there have been no studies of gut or fat body specific loss of translation machinery components, which would determine the significance of the reduced translation in these tissues for longevity.

Surprisingly, the corresponding tissues in the *Irs1*^*−/−*^ mice, the small intestine, muscle and liver, showed no reduction of polysomes, although we cannot rule out failure to detect a response smaller than the level detectable with the sample sizes available. However, protein synthesis was significantly decreased in skeletal muscle (*ex vivo*). Thus, in mice translation initiation and elongation may also be regulated by IIS, resulting in decreased protein synthesis. Our results are in line with recent findings on mTOR regulation of translation. Direct inhibition of mTOR by rapamycin injection acutely reduced polysome formation in mouse livers, but after prolonged exposure to rapamycin, polysomes returned to wild type levels^33^. Likewise, mice with a null mutation in *ribosomal protein S6 kinase*, a key regulator of translation and downstream target of mTOR and IIS, also showed no differences in polysome profile^33^. Unfortunately, incorporation of radiolabeled amino acids was not determined in this study, so it is impossible to say whether the lifespan extension seen in these mutants is truly not accompanied by downregulation of translation. Together with our findings, these results suggest that mechanisms exist in mammals to maintain the balance between translation initiation and elongation during long-term low IIS pathway and mTOR pathway activity. What these mechanisms are and to what extent they overlap for IIS and mTOR remains to be elucidated.

Reduced protein synthesis was not observed upon muscle-specific loss of *Irs1*, indicating that reduced IIS does not directly decrease translation in the muscle. Surprisingly, liver specific loss of *Irs1* decreased protein synthesis in the muscle, although not to the same extent as in the *Irs1*^*−/−*^ mice. This suggests that translation in the skeletal muscle depends, at least in part, on changes in liver metabolism, which are possibly communicated through signaling molecules.

The tissue-specific response to reduced IIS in *Drosophila* suggest that in *Irs1*^*−/−*^ mutant mice the small intestine and liver would show the greatest down-regulation of translation, however, we observed no regulation of translation in the intestine and only a trend in the liver. Such discrepancies may result from the difficulty of directly comparing tissues between different species, for example, adult insects, such as *Drosophila*, are largely post-mitotic, while mammalian tissues, as in the mouse, are proliferative, and, in the case of the muscle, potentially grow throughout life. Furthermore, the absence of yolk protein production in mammalian intestine and liver represents a fundamental difference from their worm and insect counterparts. In Snell dwarf mice, translation has been previously reported to be down regulated in the muscle^10^, suggesting that muscle translation is important for lifespan regulation. Skeletal muscle makes up a large proportion of the lean mass in mammals and regulation in this tissue may have a large effect on the organism as a whole. The liver was also reported as a site of reduced translation, though to a lesser extent than the muscle^10^. We also observed a trend suggesting decreased translation in the liver of *Irs1* mutant mice. Interestingly, reduced protein turnover in the liver was shown to correlate with lifespan extension in a study comparing multiple long-lived mouse models^34^. Unfortunately, similar data for the muscle are not available.

In conclusion, reduced translation is a phenotype of reduced IIS that is conserved from *C. elegans* to *Drosophila* and mice. Reduced translation occurs in multiple models of longevity and across multiple species. Whether translation directly regulates lifespan in each of these scenarios and what the exact mechanisms are remains to be investigated. Reduced translation is thought to exert its effect on lifespan through reducing proteotoxic stress and shifting translation to specifically synthesizing beneficial proteins, but the balance between these mechanisms and the identity of the beneficial proteins translated may vary between species, tissues and longevity models.

## Materials and Methods

### Fly stocks and fly husbandry

All mutant chromosomes were backcrossed into a *white* Dahomey (*w*^*Dah*^) strain genetic background for at least 8 generations. Fly stocks were maintained at 25 °C on a 12 h light and 12 h dark cycle and fed a standard sugar/yeast/agar diet^35^. All experimental flies were once mated females, and reared at controlled larval densities. Adult flies were kept in SYA food vials (25 flies per vial) and aged 10 d prior to dissection in cold phosphate buffered saline (PBS) and directly frozen on dry ice. Dissection of *Drosophila* gut included malpighian tubules.

### Ethics statement

This study was performed in strict accordance with the recommendations and guidelines of the Federation of European Laboratory Animal Science Associations (FELASA). The protocol was approved by the “Landesamt fuer Natur, Umwelt und Verbraucherschutz Nordrhein-Westfalen”.

### Mouse models and husbandry

All mice were maintained at 22 °C under a 12-h light/dark cycle (lights on from 7:00 AM–7:00 PM). Mice were housed in groups of three to five same-sex littermates under specific pathogen-free conditions within individually ventilated cages (Techniplast UK Ltd, Kettering, Northamptonshire, UK). Mice had ad libitum access to normal chow [ssniff® R/M-H phytoestrogen-poor (9% fat, 34% protein, 57% carbohydrate) ssniff Spezialdiäten GmbH, Soest, Germany] and water. Mice were dissected and tissues were snap-frozen in liquid-nitrogen. *Irs*^*−/−*^ and control mice were sacrificed at 74 weeks. Tissue-specific knockout mice were sacrificed at 24–25 weeks. *Irs1* KO mice were generated previously^36^, and kindly provided by Dr. Dominic Withers. *Irs* KO mice were then backcrossed for 4 generations from C57BL/6J to a C57BL/6N.

### Generation of *Irs1* conditional KO and tissue-specific KO mice

The targeting vector for disruption of *Irs1* in ES cells (dervived from C57BL/6N mice) was generated using BAC clones from the Taconic Artemis C57BL/6J RPCIB-731 BAC library. To generate a conditional *Irs1* knockout (KO) allele, exon 1 (along with 2 Kb of the upstream promoter region) of the *Irs1* locus were flanked by loxP sites. The puromycin resistance marker (PuroR) and neomycin resistance marker (NeoR) were flanked by F3 and FRT sites respectively, and the conditional KO allele was achieved after Flp-mediated removal of the selection markers. For tissue-specific KO of *Irs1*, *Irs1*^*loxP/loxP*^ mice were crossed with mice expressing Cre-recombinase under the control of the mouse albumin enhancer and promoter and the mouse alpha-fetoprotein enhancers (*Alfp*Cre mice) (Kellendonk *et al.*, 2000) or the control of creatine kinase promotor (*Ckmm*Cre)^37^. Breeding *Irs1*^*loxP/loxP*^ *Alfp*Cre mice with *Irs1*^*loxP/loxP*^, produced mice with hepatocyte specific *Irs1* deletion (denoted as *Alfp*Cre::*Irs*^*fl/fl*^) and littermate controls, whilst breeding *Irs1*^*loxP/loxP*^ *Ckmm*Cre mice with *Irs1*^*loxP/loxP*^ produced mice with muscle specific *Irs1* deletion (denoted as *Ckmm* Cre::*Irs*^*fl/fl*^) and littermate controls. Generation of conditional *Irs1* KO and tissue-specific KO mice is illustrated in Fig. S4.

### Polysome profiling

Polysome profiles were performed as previously described with minor modifications^38^. Tissues were homogenized on ice in 700–1200 μl polysome extraction buffer (300 mM NaCl, 50 mM Tris-HCL (pH 8.0), 10 mM MgCl2, 1 mM, EGTA, 200 mg heparin/ml, 400 U RNAsin/ml, 1.0 mM, phenylmethylsulfonyl fluoride, 0.2 mg cycloheximide/ml, 1% Triton X-100, 0.1% Sodium deoxycholate). Lysates were mixed and left on ice for 10 min. Debris was the removed by spinning at 9,000 g (4 °C) for 10 min and equal A260 units (5–10) for *Drosophila* or mg of protein (1–4 mg) for mouse samples of the supernatant was layered onto a sucrose gradient, 10–50% for *Drosophila* tissues and 17–50% for mouse tissues, in high salt resolving buffer (140 mM NaCl, 25 mM Tris-HCL (pH 8.0), 10 mM MgCl2). Monosomes, polysomes and ribosomal subunits were separated using a Beckman SW41Ti rotor (38,000 rpm at 1.5 h, 4 °C). Profiles were continuously monitored (Ab 252 nm) using a Teledyne density gradient fractionator. Data was collected using a Dataq DI-148-U device and recorded using WinDaq XL.

### ^35^S-methionine/^35^S-cysteine incorporation assay

Our method for determining *ex-vivo* incorporation of radio-labeled amino acids is based on several published protocols^39^,^40^,^41^. Briefly, tissues of both *Drosophila* and mice were dissected and collected in DMEM (#41965-047, Gibco) without any supplements, at room temperature. Where necessary, experiments were performed in batches to avoid long handling time. For *Drosophila*, 5–8 tissues per sample were used. For mice, tissues were cut into pieces of approximately 1 mm^3^ while in a petridish filled with DMEM. 2–3 pieces of tissue were used per sample and 4–5 technical replicates were performed for each individual tissue. Mouse tissues in DMEM were placed in a 12-well plate in a cell culture incubator at 37 °C and 5%CO_2_ for 30 min prior to labeling.

For labeling, DMEM was replaced with methionine and cysteine free DMEM (#21-013-24, Gibco), supplemented with ^35^S-labeled methionine and cysteine (#NEG772, Perkin-Elmer). Drosophila tissues were incubated in uncapped eppendorf tubes on a shaking platform at room temperature; mouse tissue sections were placed in a cell culture incubator. After 60 min, samples were placed on ice, washed in ice cold PBS and lysed in RIPA buffer (150 mM sodium chloride, 1.0% NP-40, 0.5% sodium deoxycholate, 0.1% SDS, 50 mM Tris, pH 8.0) using a pestle gun (VWR, Germany). Lysates were cleared by centrifugation at 13.000 rpm, 4 °C for 10 min. Protein was precipitated by adding 1 volume of 20% TCA, incubating for 15 min on ice and centrifugation at 13.000 rpm, 4 °C for 15 min. The pellet was washed twice in acetone and resuspended in 200 ul of 4 M guanine-HCl. The samples were briefly spun to avoid measuring any non-dissolved radioactive particles. Half the sample was added to 10 ml of scintillation fluid (Ultima Gold, Perkin-Elmer) and counted for 5 min per sample in a scintillation counter (Perkin-Elmer). The remaining sample was used to determine protein content using BCA (Pierce), following the manufacturers protocol. Scintillation counts were then normalized to total protein content prior to statistical analysis.

### q-RT-PCR

RNA was isolated using TRIzol (Thermo-Fisher), treated with DNAse (Qiagen) and purified by isopropanol precipitation. cDNA was prepared using the RETROscript reverse transcription kit (Ambion) as per manufacturers instructions. Taqman probes against Irs1 and beta2-microglobulin were obtained from Applied Biosystems and run on a 7900HT real-time PCR system.

### Statistics

Area under the curve for polysome profiles were calculated using an R script developed in house (https://paul-essers.shinyapps.io/ShinyProfile/). For pairwise comparisons of the AUCs of the polysome profiles, student t-tests were carried out in R. For cross-experiment comparisons of the mouse small intestine and liver polysome profiles, two-way ANOVAs were performed in R, in order to account for variation in experimental date and age. For analysis of ^35^S-methionine incorporation experiments, student t-tests or ANOVA were carried out in R. In all cases where a student t-test was performed, homoscedasticity was tested to determine whether standard deviations should be pooled. P-values for all variance and t-tests are shown in Supplementary Table 1.

## Additional Information

**How to cite this article**: Essers, P. *et al.* Reduced insulin/insulin-like growth factor signaling decreases translation in *Drosophila* and mice. *Sci. Rep.* **6**, 30290; doi: 10.1038/srep30290 (2016).

## Supplementary Material

Supplementary Information
