# Supplementary material for: Reduced insulin/insulin-like growth factor signaling decreases translation in Drosophila and mice
Source: Sci Rep. 2016 Jul 25;6:30290. doi: 10.1038/srep30290 (PMC4959029; doi:10.1038/srep30290)
Supplement: Supplementary Information [file srep30290-s1.pdf]

Supplementary Information

Reduced insulin/insulin growth factor Signaling decreases translation in Drosophila and mice

Paul Essers1#, Luke S. Tain1#, Tobias Nespital1, Joana Goncalves1, Jenny Froehlich1, and Linda Partridge1, 2*

Figure S1

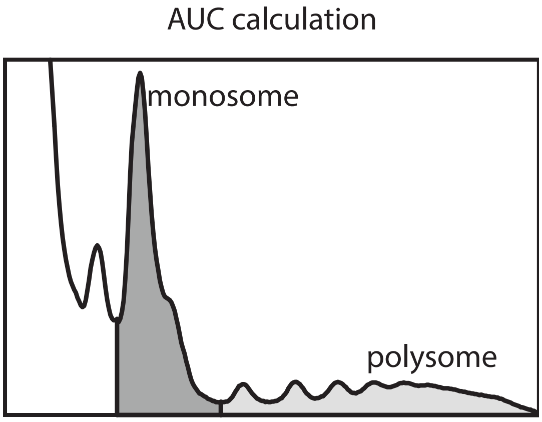

Figure S2

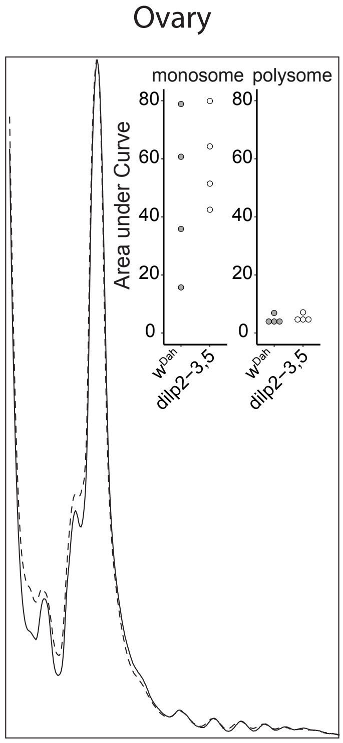

Ovary
monosome
polysome
Area under Curve
80
60
40
20
0
w^Dah
dilp2−3,5

Figure S3

a Liver

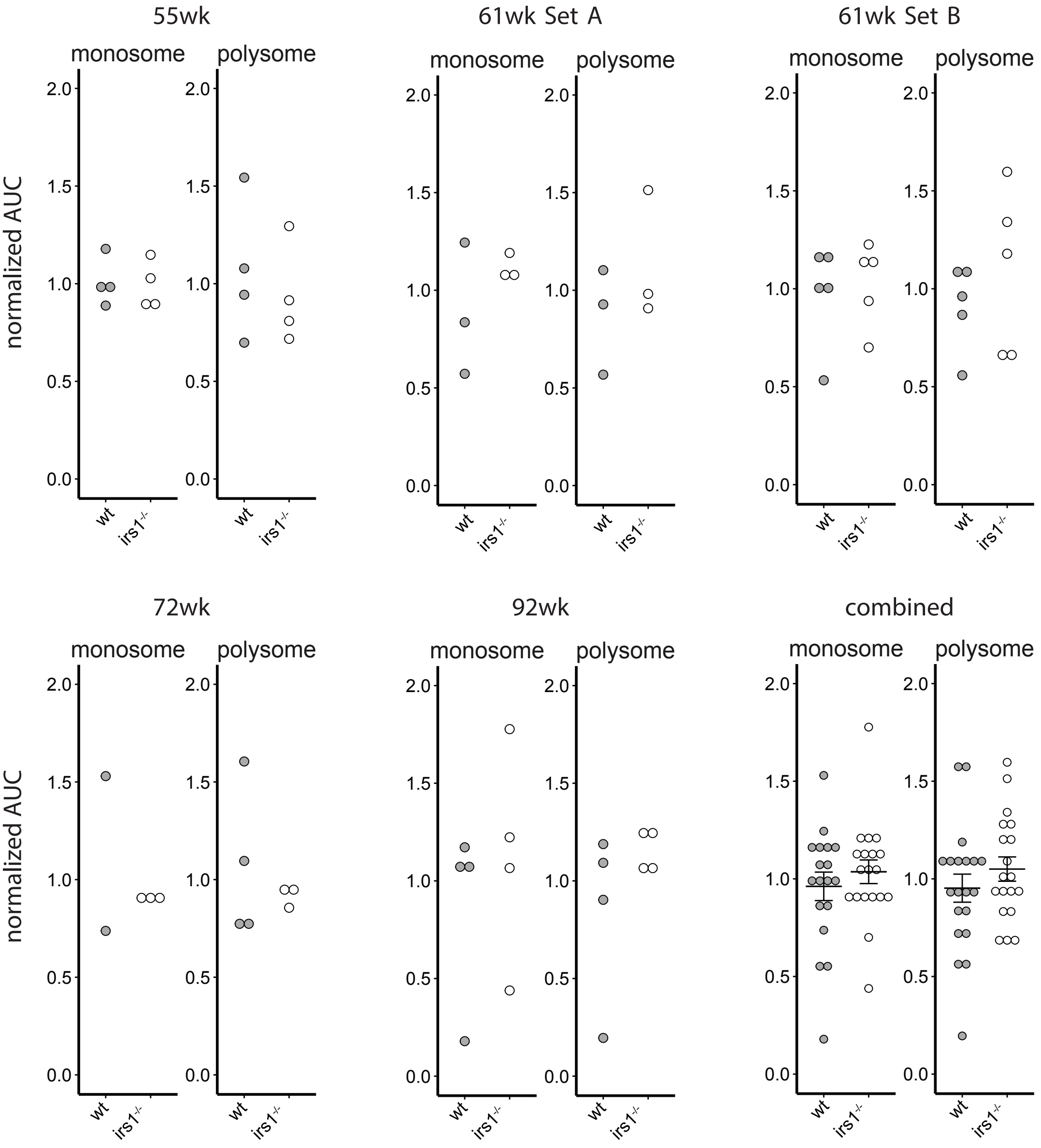

b Small Intestine

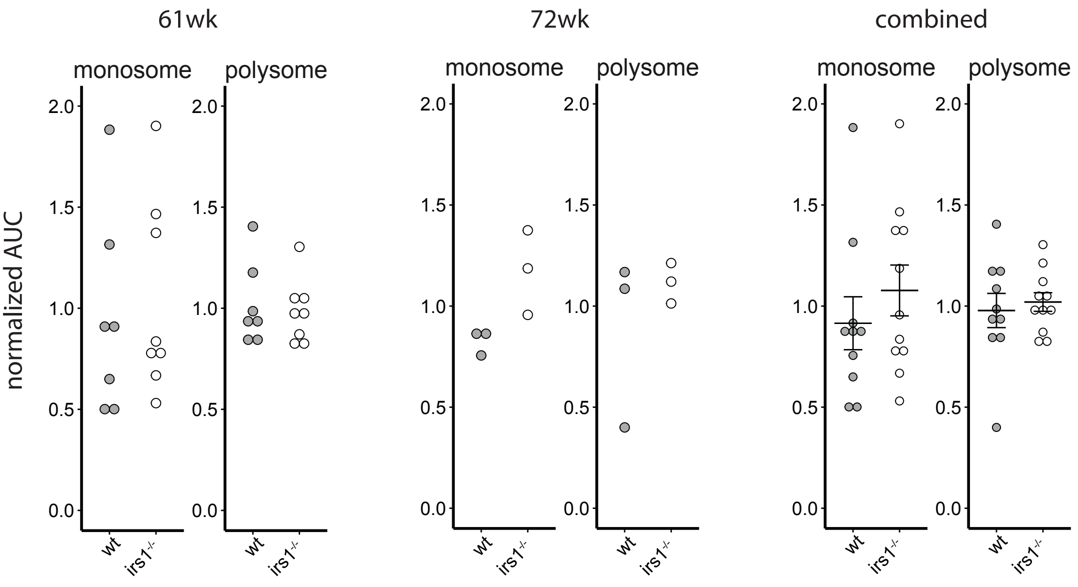

Figure S4

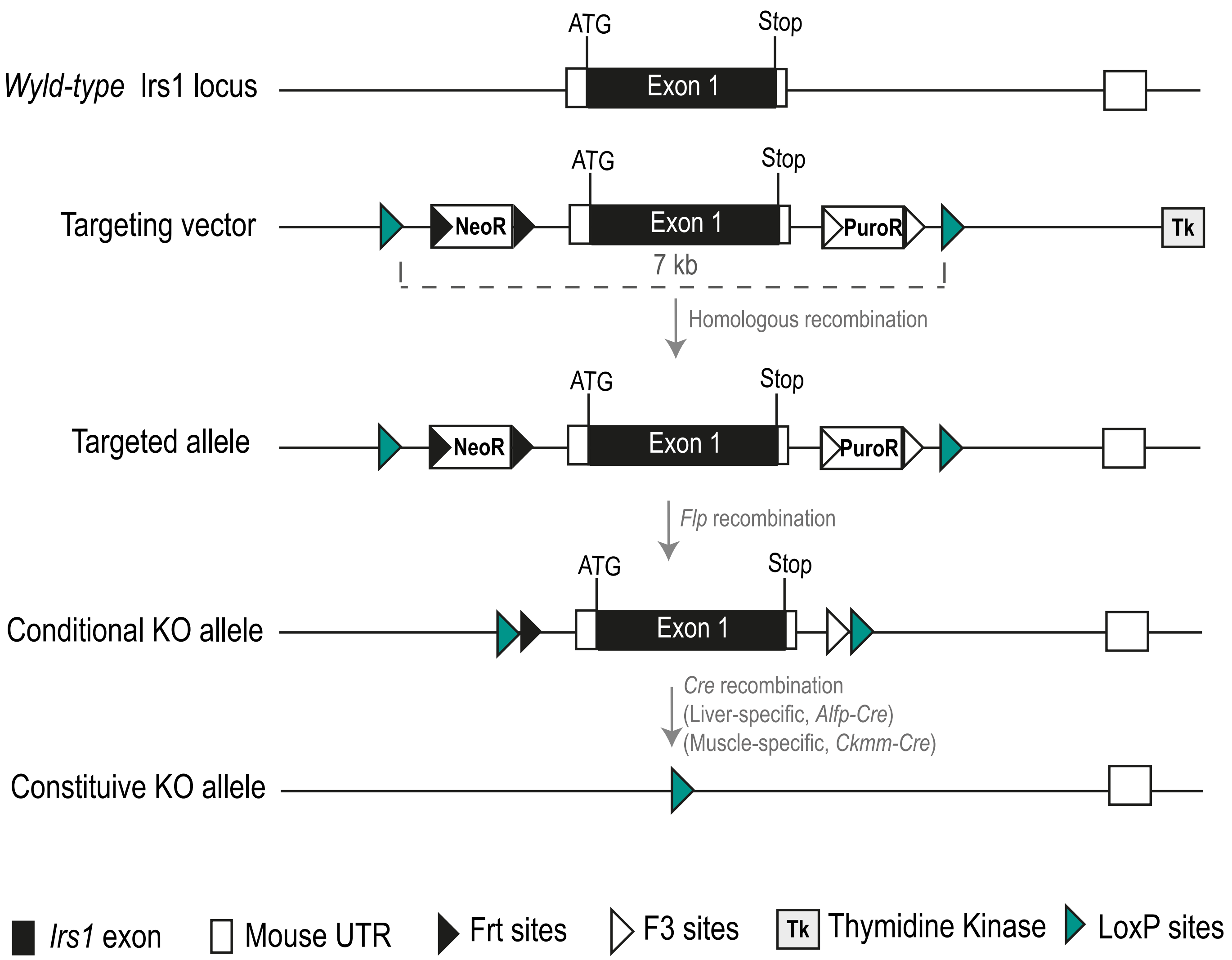

Wyld-type Irs1 locus
ATG
Stop
Exon 1
Targeting vector
NeoR
PuroR
Tk
7 kb
Homologous recombination
Targeted allele
Flp recombination
Conditional KO allele
Cre recombination
(Liver-specific, Alfp-Cre)
(Muscle-specific, Ckmm-Cre)
Constituive KO allele
Irs1 exon
Mouse UTR
Frt sites
F3 sites
Thymidine Kinase
LoxP sites

Figure S5

a

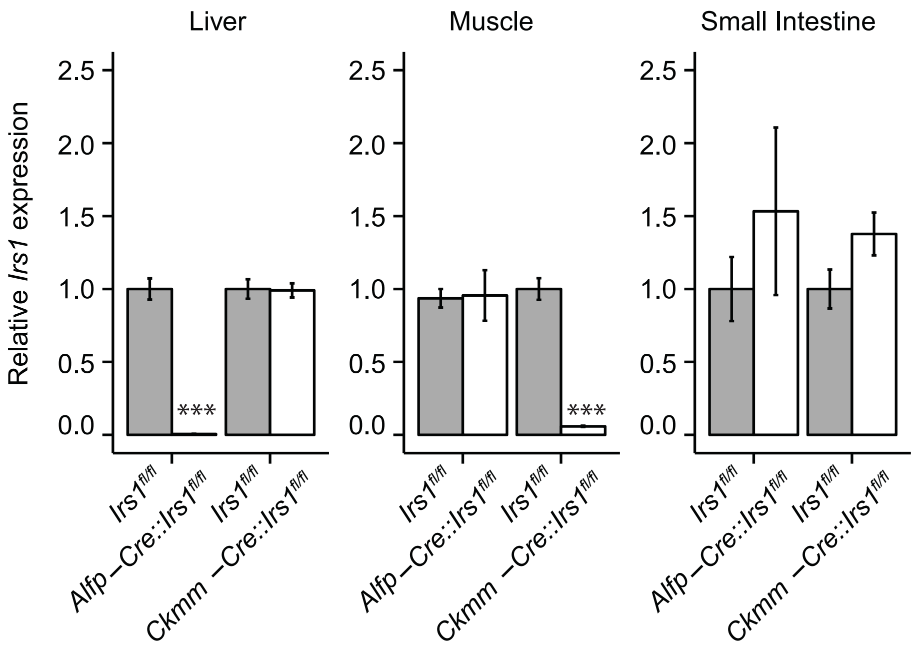

Liver
Muscle
Small Intestine
Relative Irs1 expression
2.5
2.0
1.5
1.0
0.5
0.0
***
***
Irs1fl/fl
Alfp−Cre::Irs1fl/fl
Irs1fl/fl
Ckmm −Cre::Irs1fl/fl

Drosophila

Genotype comparison	Figure #	Polysome profiles	Fraction	f.test	t.test	n (dilp/wDah)
dilp2-3,5 -/- vs wDah	1a	Thorax	monosome	0.2489236	0.0426719	3/3
dilp2-3,5 -/- vs wDah	1a	Thorax	polysome	0.8302394	0.784612	3/3
dilp2-3,5 -/- vs wDah	1a	Fatbody	monosome	0.5342847	0.24601	4/4
dilp2-3,5 -/- vs wDah	1a	Fatbody	polysome	0.0578441	0.0153141	4/4
dilp2-3,5 -/- vs wDah	1a	Gut	monosome	0.9933999	0.4287569	3/4
dilp2-3,5 -/- vs wDah	1a	Gut	polysome	0.3773318	0.0188145	3/4
dilp2-3,5 -/- vs wDah	1a	Gut	monosome	0.4046775	0.4986104	4/4
dilp2-3,5 -/- vs wDah	1a	Gut	polysome	0.8409396	0.579644	4/4

Genotype comparison		35S incorporation assay		f.test	t.test	n (dilp/wDah)
dilp2-3,5 -/- vs wDah	1b	Thorax		0.0053	0.9931	17/17
dilp2-3,5 -/- vs wDah	1b	Fatbody		0.1047	0.0123	37/37
dilp2-3,5 -/- vs wDah	1b	Gut		0.1946	0.5593	37/37

Mouse

Genotype comparison		Polysome profiles	Fraction	f.test	t.test	n (Irs/wt)
Irs1-/- vs control	2a	Liver	monosome	0.0028	0.1889	4/3
Irs1-/- vs control	2a	Liver	polysome	0.0554	0.5474	4/3
Irs1-/- vs control	2a	Muscle	monosome	0.5152	0.2444	4/3
Irs1-/- vs control	2a	Muscle	polysome	0.2181	0.8466	4/3
Irs1-/- vs control	2a	Small Intestine	monosome	0.1710	0.0525	3/3
Irs1-/- vs control	2a	Small Intestine	polysome	0.1063	0.4084	3/3

Genotype comparison		Polysome profiles	Fraction	f.test	t.test	n (Irs/wt)
Irs1-/- vs control	S3b	Liver	monosome	0.7830	0.9034	19/20
Irs1-/- vs control	S3b	Liver	polysome	0.6548	0.4002	19/20
Irs1-/- vs control	S3b	Small Intestine	monosome	0.9933	0.3830	11/10
Irs1-/- vs control	S3b	Small Intestine	polysome	0.0967	0.6694	11/10

Genotype comparison		35S incorporation assay		f.test	t.test	n (Irs/wt)
Irs1-/- vs control	2b	Liver		0.0674	0.1315	4/3
Irs1-/- vs control	2b	Muscle		0.0720	0.0157	4/3
Irs1-/- vs control	2b	Small Intestine		0.0636	0.9979	3/3

Genotype comparison		Polysome profiles	Fraction	f.test	t.test	n (Cre+/Cre-)
Ckmm-Cre::Irs1 fl/fl vs Irs1 fl/fl	3a	Liver	monosome	0.7901	0.0015	4/4
Ckmm-Cre::Irs1 fl/fl vs Irs1 fl/fl	3a	Liver	polysome	0.8631	0.6275	4/4
Ckmm-Cre::Irs1 fl/fl vs Irs1 fl/fl	3a	Muscle	monosome	0.9254	0.3305	3/3
Ckmm-Cre::Irs1 fl/fl vs Irs1 fl/fl	3a	Muscle	polysome	0.0528	0.6977	3/3
Ckmm-Cre::Irs1 fl/fl vs Irs1 fl/fl	3a	Small Intestine	monosome	0.8313	0.1536	4/4
Ckmm-Cre::Irs1 fl/fl vs Irs1 fl/fl	3a	Small Intestine	polysome	0.3122	0.3265	4/4

Genotype comparison		35S incorporation assay		f.test	t.test	n (Cre+/Cre-)
Ckmm-Cre::Irs1 fl/fl vs Irs1 fl/fl	3b	Liver		paired	0.2226	4/4
Ckmm-Cre::Irs1 fl/fl vs Irs1 fl/fl	3b	Muscle		paired	0.7872	4/4
Ckmm-Cre::Irs1 fl/fl vs Irs1 fl/fl	3b	Small Intestine		paired	0.5622	4/4

Genotype comparison		Polysome profiles	Fraction	f.test	t.test	n (Cre+/Cre-)
Alfp-Cre::Irs1 fl/fl vs Irs1 fl/fl	3a	Liver	monosome	0.7745	0.0185	4/4
Alfp-Cre::Irs1 fl/fl vs Irs1 fl/fl	3a	Liver	polysome	0.7183	0.0575	4/4
Alfp-Cre::Irs1 fl/fl vs Irs1 fl/fl	3a	Muscle	monosome	0.0415	0.6429	3/3
Alfp-Cre::Irs1 fl/fl vs Irs1 fl/fl	3a	Muscle	polysome	0.5510	0.9111	3/3
Alfp-Cre::Irs1 fl/fl vs Irs1 fl/fl	3a	Small Intestine	monosome	0.3668	0.0197	4/4
Alfp-Cre::Irs1 fl/fl vs Irs1 fl/fl	3a	Small Intestine	polysome	0.2128	0.8925	4/4

Genotype comparison		35S incorporation assay		f.test	t.test	n (Cre+/Cre-)
Alfp-Cre::Irs1 fl/fl vs Irs1 fl/fl	3c	Liver		0.7915	0.6695	4/4
Alfp-Cre::Irs1 fl/fl vs Irs1 fl/fl	3c	Muscle		0.9979	0.0006	4/4
Alfp-Cre::Irs1 fl/fl vs Irs1 fl/fl	3c	Small Intestine		1.0000	0.1156	4/4

Repeat expt.

Genotype comparison		35S incorporation assay		f.test	t.test	n
Alfp-Cre::Irs1 fl/fl vs Irs1 fl/fl	3d	Liver		paired	0.587117	4/4
Alfp-Cre::Irs1 fl/fl vs Irs1 fl/fl	3d	Muscle		paired	0.0411963	4/4
Alfp-Cre::Irs1 fl/fl vs Irs1 fl/fl	3d	Small Intestine		paired	0.0382622	4/4

Fig S1. Explanation of area under curve calculations. Profiles were aligned by their lowest point or, if the profile did not drop to a baseline before the end of the gradient, by the valley preceding the first polysome. Differences in A254 absorption measurements between the lowest point and the profile were then summed over the monosome and polysome to generate AUC values.

Fig S2. Polysome formation is unaltered in the ovaries of dilp2-3,5 mutants. (A) Representative polysome profiles of isolated 10 day old Drosophila ovaries. Insets show the area under the curve measurements for monosomes and combined polysomes.

Fig S3. Additional mouse polysome profiles. (A) Area under the curve measurements of polysome profiles of dissected mouse tissues, respective ages shown. Polysome profiles were performed on samples collected from mice from independent breedings to verify the results in Fig 2a. All values were normalized to batch average. Unpaired student t-test was used to establish significance. No differences were detected between control and Irs1-/- polysome profiles in either liver or small intestine.

Fig S4. Generation of Irs1 conditional KO and tissue-specific KO mice. Targeting strategy for the disruption of the Irs1 gene in mice, as described in the materials and methods.

Fig S5. Characterization and polysome profiles of tissue-specific Irs1 knockouts. (A) q-RT-PCR of Irs1 transcripts in tissues of Cre::Irs1fl/fl and Alfp-Cre::Irs1fl/fl compared to controls. *** $p < 0.001$.

Table S1. Summary of statistics. For each comparison, an F-test was performed to determine whether the variances were equal between conditions. Variances were considered equal at $p > 0.05$. Subsequently, a t-test was performed assuming either equal variances or unequal variances, as determined by the F-test.
